# Multi-cohort shotgun metagenomic analysis of oral and gut microbiota overlap in healthy adults

**DOI:** 10.1038/s41597-024-02916-x

**Published:** 2024-01-16

**Authors:** Armin Rashidi, Hakan Gem, Jeffrey S. McLean, Kristopher Kerns, David R. Dean, Neelendu Dey, Samuel Minot

**Affiliations:** 1 Clinical Research Division, Fred Hutchinson Cancer Center, Seattle, WA USA; 2 Division of Oncology, Department of Medicine, University of Washington, Seattle, WA USA; 3 School of Dentistry, University of Washington, Seattle, WA USA; 4 Division of Gastroenterology, Department of Medicine, University of Washington, Seattle, WA USA; 5 Microbiome Research Initiative, Vaccine and Infectious Disease Division, Fred Hutchinson Cancer Center, Seattle, WA USA

**Keywords:** Microbiome, Metagenomics

## Abstract

The multitude of barriers between the mouth and colon may eliminate swallowed oral bacteria. Ascertaining the presence of the same bacteria in the mouth and colon is methodologically challenging partly because 16S rRNA gene sequencing – the most commonly used method to characterize the human microbiota – has low confidence in taxonomic assignments deeper than genus for most bacteria. As different species of the same genus can have low-level variation across the same 16S rRNA gene region, shotgun sequencing is needed to identify a true overlap. We analyzed a curated, multi-cohort, shotgun metagenomic database with species-level taxonomy and clade-specific marker genes to fill this knowledge gap. Using 500 paired fecal/oral (4 oral sites) samples from 4 healthy adult cohorts, we found a minute overlap between the two niches. Comparing marker genes between paired oral and fecal samples with species-level overlap, the pattern of overlap in only 7 individuals was consistent with same-strain colonization. These findings argue against ectopic colonization of oral bacteria in the distal gut in healthy adults.

## Introduction

Several barriers separate the oral and colonic microbiota in healthy adults. These barriers include gastric acid, bile salts, mucosal immunoglobulins, antimicrobial peptides, colonic hypoxia, fecal toxins, and microbiota-mediated colonization resistance. How effectively these barriers prevent colonization of oral bacteria in the colon despite the anatomic connection between the two habitats allowing the passage of approximately 10^11^ oral bacteria per day via swallowing saliva^1^ remains debated^2–8^. One reason for the inconsistency of the results from different studies is the use of different sequencing platforms, some (*e.g*., those based on short amplicon sequencing and operational taxonomic units) yielding a lower resolution of taxonomic classification and potentially overestimating the oral/colonic microbiota overlap, while others (*e.g*., amplicon sequence variants and shotgun sequencing) providing a higher resolution. Finally, finding low-abundance species in saliva – the component chosen by most previous studies due to the ease of collection – through shotgun sequencing is challenging because of high-level contamination by host DNA, leading to low coverage depth^9^. This limitation may contribute to the underestimation of the oral/colonic microbiota overlap.

The presence of a true, strain-level overlap between the two niches would indicate that the oral flora directly contributes microbiota to the gut flora. This would be opposite to a scenario in which distinct pools of niche-adapted microbiota inhabit the two sites. Distinguishing between these two scenarios is clinically important because ectopically colonized oral bacteria in the first scenario may cause pathology (*e.g*., intestinal inflammation). As an example, colonization of the distal gut with *Haemophilus parainfluenzae* or *Veillonella parvula* of likely oral origin has been associated with inflammatory bowel disease and its severity/activity^10–14^.

To address this knowledge gap, we used publicly available curatedMetagenomicData^15^ to access uniformly processed, integrated shotgun metagenomic sequencing data from >5,000 samples from multiple body sites from >25 studies with standardized per-participant metadata. These studies span both health and disease states and were done in >25 countries. We mined this large, diverse dataset to identify paired stool and oral (any site) samples and investigate the extent of oral/colonic microbiota overlap in healthy adults.

## Results

### Saliva-Stool microbiota overlap

**Fig. 1:**
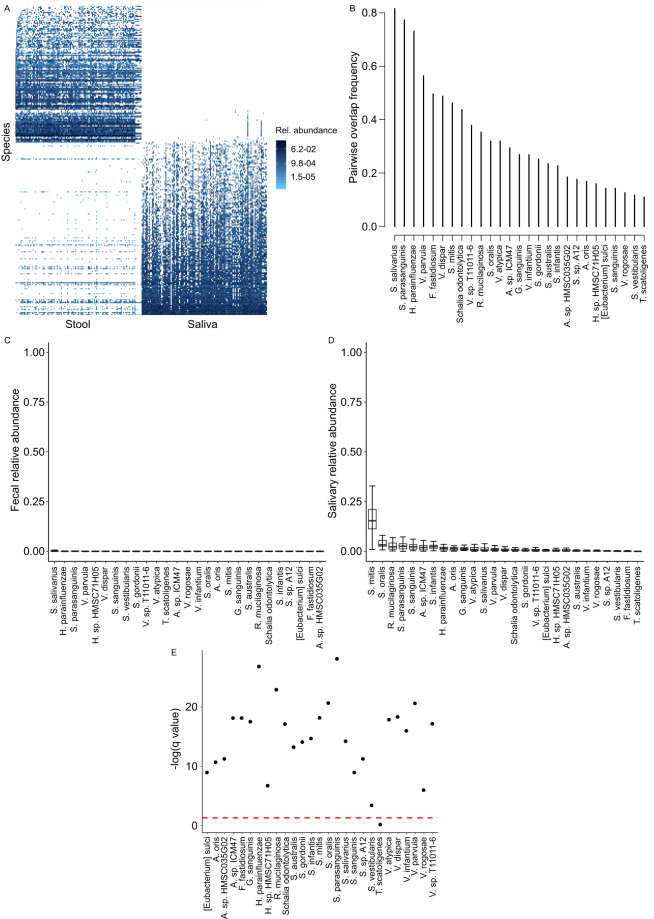
Overlap between salivary and fecal microbiota. (**A**) Species-level microbiota heatmap. Bray-Curtis distance and non-metric multidimensional scaling were used for ordination, and taxa abundances were log transformed. Samples were sorted along the x-axis according to sample type for easy visualization. Relative abundances are color coded, with darker colors indicating greater abundances. (**B**) Pairwise overlap frequency for species overlapping the two sample types in ≥10% of sample pairs. (**C** and **D**) Fecal and oral relative abundances of the overlapping species in (**B**). Boxplots show the median (midline), interquartile range (box boundaries), and non-outlier maximum and minimum (whiskers). (**E**) Comparison of fecal vs. oral abundance for each overlapping species. The red line shows statistical significance threshold (q < 0.05 from a Wilcoxon test after correction of p values for multiple testing). Points above this line are statistically significant.

Samples included in this dataset included 121 pairs (116 pairs from study #2 and 5 from study #1) (Fig. 1A). Twenty-seven species overlapped between stool and saliva in ≥10% of the pairs, with the 3 most frequently overlapping species (>70% of the pairs) being *Streptococcus salivarius*, *Streptococcus parasanguinis*, and *Haemophilus parainfluenzae*. Several other *Streptococcus* and *Veillonella* species dominated the remainder of the overlapping list (Fig. 1B). The fecal relative abundance of the overlapping species was very low, with medians <0.1% (Fig. 1C). With 1 exception, these abundances were significantly lower than corresponding oral abundances (q < 0.05, Fig. 1C–E), supporting an oral origin. The only exception, where the difference in abundance between the two niches did not reach statistical significance, was *Tractidigestivibacter scatoligenes*. This species had only ~10% pairwise overlap frequency.

### Buccal mucosa-Stool microbiota overlap

**Fig. 2:**
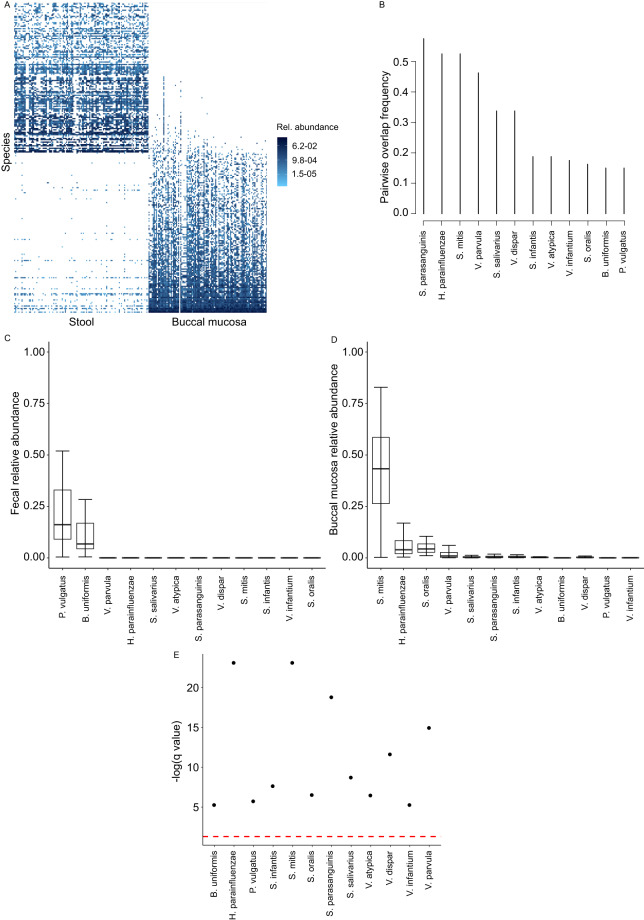
Overlap between buccal mucosa and fecal microbiota. (**A**) Species-level microbiota heatmap. Bray-Curtis distance and non-metric multidimensional scaling were used for ordination, and taxa abundances were log transformed. Samples were sorted along the x-axis according to sample type for easy visualization. Relative abundances are color coded, with darker colors indicating greater abundances. (**B**) Pairwise overlap frequency for species overlapping the two sample types in ≥10% of sample pairs. (**C,D**) Fecal and oral relative abundances of the overlapping species in (**B**). Boxplots show the median (midline), interquartile range (box boundaries), and non-outlier maximum and minimum (whiskers). (**E**) Comparison of fecal vs. oral abundance for each overlapping species. The red line shows statistical significance threshold (q < 0.05 from a Wilcoxon test after correction of p values for multiple testing). Points above this line are statistically significant.

This dataset included 112 pairs, all from study #1 (Fig. 2A). Twelve species overlapped between stool and buccal mucosa in ≥10% of the pairs, with the 3 most frequently overlapping species (>50% of the pairs) being *S. parasanguinis*, *H. parainfluenzae*, and *S. mitis*. Several other *Streptococcus* and *Veillonella* species dominated the remainder of the overlapping list (Fig. 2B). Two overlapping species, *Phocaeicola vulgatus* and *B. uniformis*, had a relatively high fecal relative abundance (medians 5–15%) (Fig. 2C), but trace buccal mucosal relative abundance (median <0.1%, q < 0.05) (Fig. 2D), suggesting a fecal origin. The fecal relative abundance of the other overlapping species was very low, with medians <0.1% (q < 0.05 compared to their oral abundance; Fig. 2C–E) and supporting their oral origin.

### Supragingival plaque-Stool microbiota overlap

**Fig. 3:**
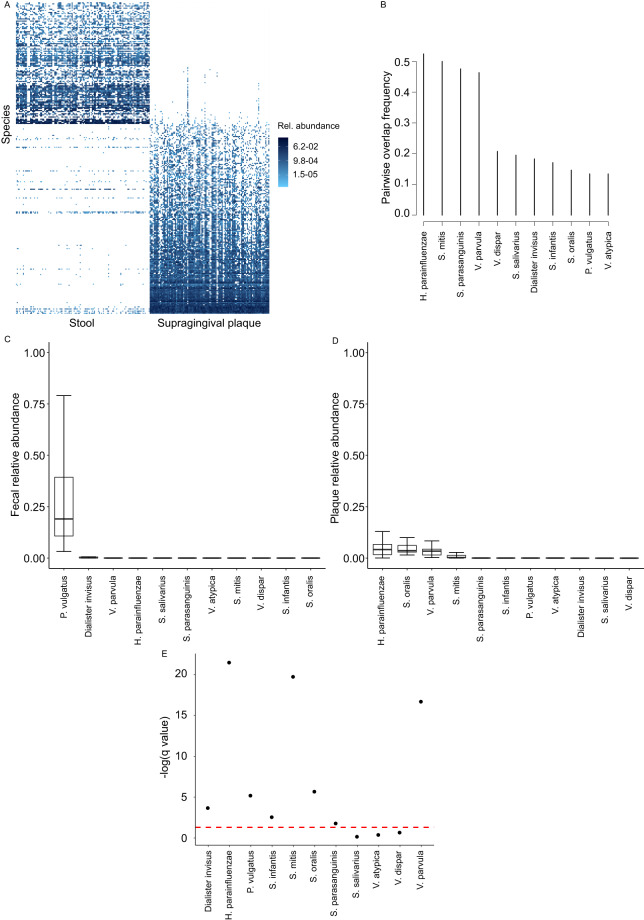
Overlap between supragingival plaque and fecal microbiota. (**A**) Species-level microbiota heatmap. Bray-Curtis distance and non-metric multidimensional scaling were used for ordination, and taxa abundances were log transformed. Samples were sorted along the x-axis according to sample type for easy visualization. Relative abundances are color coded, with darker colors indicating greater abundances. (**B**) Pairwise overlap frequency for species overlapping the two sample types in ≥10% of sample pairs. (**C,D**) Fecal and oral relative abundances of the overlapping species in (**B**). Boxplots show the median (midline), interquartile range (box boundaries), and non-outlier maximum and minimum (whiskers). (**E**) Comparison of fecal vs. oral abundance for each overlapping species. The red line shows statistical significance threshold (q < 0.05 from a Wilcoxon test after correction of p values for multiple testing). Points above this line are statistically significant.

This dataset included 118 pairs, all from study #1 (Fig. 3A). Eleven species overlapped between stool and saliva in ≥10% of the pairs, with the 3 most frequently overlapping species (~50% of the pairs) being *H. parainfluenzae*, *S. mitis*, and *S. parasanguinis*. Several other *Streptococcus* and *Veillonella* species dominated the remainder of the overlapping list (Fig. 3B). One overlapping species, *P. vulgatus*, had a relatively high fecal relative abundance (median ~20%) (Fig. 3C), but trace supragingival plaque relative abundance (median <0.1%, q < 0.05) (Fig. 3D), suggesting a fecal origin. The fecal relative abundance of the other overlapping species was very low, with medians <0.1%. With 3 exceptions, these abundances were significantly lower than corresponding oral abundances (q < 0.05, Fig. 2C–E), supporting an oral origin. These 3 exceptions, where the difference in abundance between the two niches did not reach statistical significance, were *S. salivarius*, *V. atypica*, and *V. dispar*. These species had <20% pairwise overlap frequency.

### Tongue dorsum-Stool microbiota overlap

**Fig. 4:**
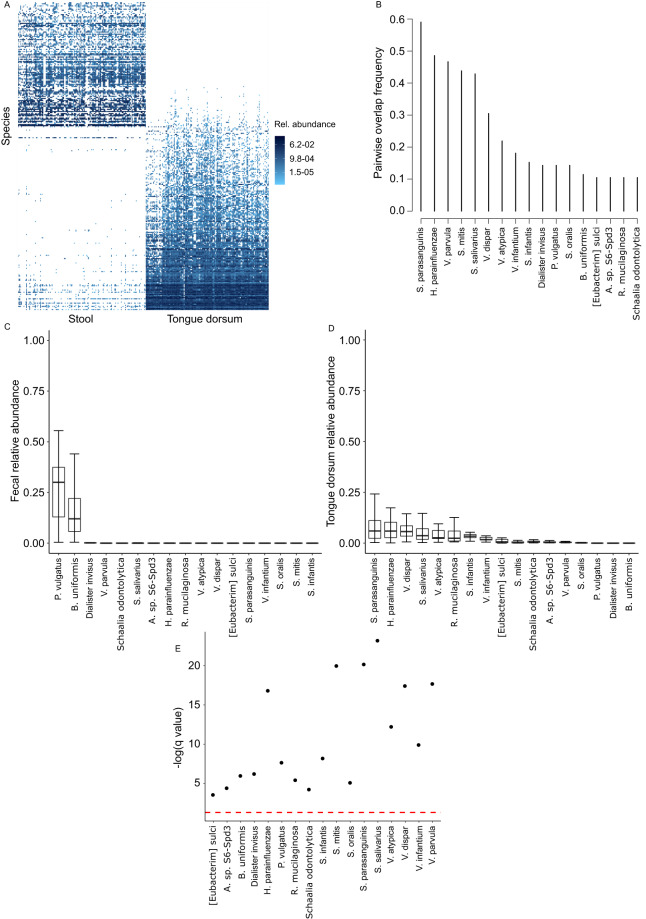
Overlap between tongue dorsum and fecal microbiota. (**A**) Species-level microbiota heatmap. Bray-Curtis distance and non-metric multidimensional scaling were used for ordination, and taxa abundances were log transformed. Samples were sorted along the x-axis according to sample type for easy visualization. Relative abundances are color coded, with darker colors indicating greater abundances. (**B**) Pairwise overlap frequency for species overlapping the two sample types in ≥10% of sample pairs. (**C,D**) Fecal and oral relative abundances of the overlapping species in (**B**). Boxplots show the median (midline), interquartile range (box boundaries), and non-outlier maximum and minimum (whiskers). (**E**) Comparison of fecal vs. oral abundance for each overlapping species. The red line shows statistical significance threshold (q < 0.05 from a Wilcoxon test after correction of p values for multiple testing). Points above this line are statistically significant.

This dataset included 149 pairs (129 pairs from study #1 and 20 from study #3) (Fig. 4A). Eighteen species overlapped between stool and tongue dorsum in ≥10% of the pairs, with the 3 most frequently overlapping species (45–60% of the pairs) being *S. parasanguinis*, *H. parainfluenzae*, and *Veillonella parvula*. Several other *Streptococcus* and *Veillonella* species dominated the remainder of the overlapping list (Fig. 4B). Two overlapping species, *P. vulgatus* and *B. uniformis*, had a relatively high fecal relative abundance (medians 10–30%) (Fig. 4C), but trace tongue dorsum relative abundance (median <0.1%, q < 0.05) (Fig. 4D), suggesting a fecal origin. The fecal relative abundance of the other overlapping species was very low, with medians <0.1% (q < 0.05 compared to their oral abundance; Fig. 4C–E) and supporting their oral origin.

### Overlap using clade-specific marker genes

**Fig. 5:**
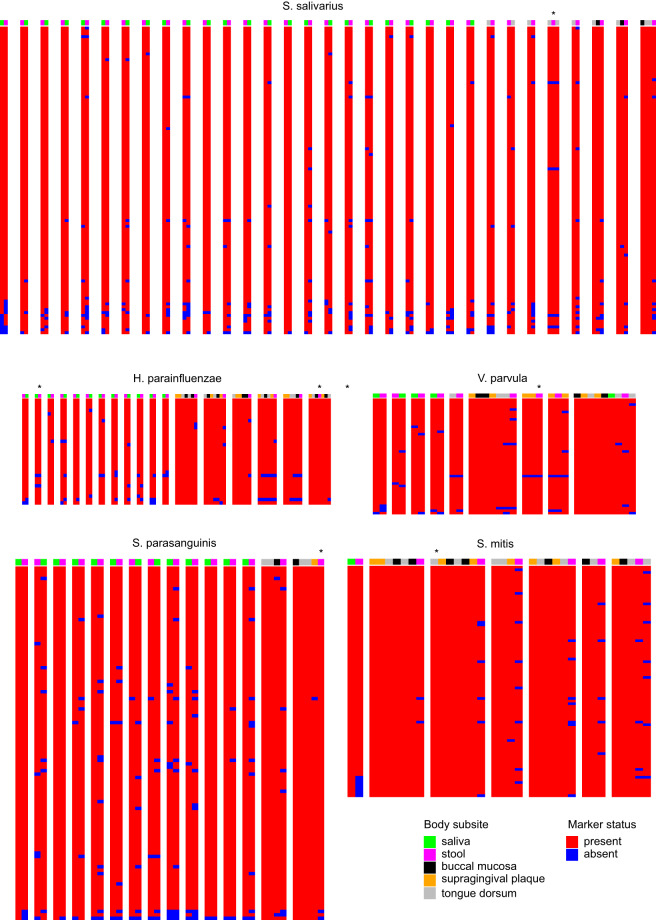
Overlap of clade-specific marker genes between fecal and oral microbiota. Unique clade-specific marker genes for five frequently overlapping species were compared between fecal and oral samples. Only samples with at least 90% coverage for marker genes of the species of interest were selected. Each bar is a sample and each set of bars shows samples from the same individual. Each row is a marker gene. Asterisks show stool samples with at least one oral sample from the same individual with the same marker gene presence/absence pattern.

Next, and considering our interest in overlapping bacteria of oral origin, we selected *S. parasanguinis*, *H. parainfluenzae*, *S. salivarius*, *S. mitis*, and *V. parvula* for clade-specific marker gene comparison between fecal and oral samples. Species-specific marker gene presence/absence patterns for within-subject samples with high coverage for these genes are visualized in Fig. 5. These patterns were consistent with same-strain colonization of stool and an oral sample in only 7 individuals: (i) *S. salivarius*: tongue dorsum (1 individual), (*ii*) *H. parainfluenzae*: saliva (1 individual); tongue dorsum and buccal mucosa (1 individual); tongue dorsum, buccal mucosa, and supragingival plaque (1 individual), (iii) *V. parvula*: supragingival plaque (1 individual), (iv) *S. parasanguinis*: saliva (1 individual); tongue dorsum and buccal mucosa (1 individual).

## Discussion

We analyzed curated shotgun metagenomics data from 500 paired stool/oral samples from 4 healthy adult cohorts. Samples were paired in that each pair consisted of a stool sample and one of the 4 oral sites (saliva, supragingival plaque, tongue dorsum, and buccal mucosa) from the same individual. First, we observed that at the species level, *S. parasanguinis*, *H. parainfluenzae*, *S. salivarius*, *S. mitis*, and *V. parvula* (members of the normal oral flora) frequently overlapped between oral and fecal microbiota, with a higher oral than fecal relative abundance, suggesting an oral origin. Next, we compared patterns of species-specific marker gene presence/absence in sample pairs with high coverage. The idea here was to take advantage of marker gene loss and gain events to identify different strains of the same species, acknowledging that this method would not distinguish between strains that are different only in single nucleotide polymorphisms within species-specific marker genes. Therefore, our approach may overestimate strain similarity but would not underestimate it. This turned out to be advantageous because marker gene comparisons showed patterns consistent with same-strain overlap between fecal and oral niches in only 7 individuals. Thus, true overlap is expected to be rare. Overall, these findings support the hypothesis that oral-fecal species overlap is driven by separate pools of niche-adapted strains of the same species rather than oral strains surviving transit to the gut in low quantities. Highly specific niche adaptation by subspecies of the same, seemingly generalist species has been demonstrated in niches distant by only a few millimeters and in communication with one another, *e.g*. different sites in the oral cavity^16^.

A powerful barrier separating the oral and gut microbiota is gastric acid, with a pH ranging between 1.5 in a fasting state and 3.0–5.0 during eating^17^. While this degree of acidity is lethal to most oral bacteria, *S. salivarius* and *H. parainfluenza* are potent urease producers in the oral cavity and can increase the pH in their immediately surrounding microenvironment through their urease activity^18,19^. This may allow a fraction of these bacteria to resist gastric acid, especially during eating, until they are passed on to the intestines. Notably, in 4 of the 7 individuals with marker gene presence/absence patterns consistent with same-strain colonization of the mouth and colon, the species were *S. salivarius* or *H. parainfluenza*.

Various methods were used in previous studies to determine the extent of ectopic gut colonization by oral bacteria in adults. Methodological variation has likely been a major contributor to the somewhat different results observed. In our recent analysis of short amplicon sequence variants (ASVs) from 66 saliva-stool sample pairs from healthy adults from two countries, we found no overlap between the two niches^7^. The only exception was *Dialister invisus*, a predominantly oral bacteria^20^. This species was present in both niches in ~25% of the subjects, but with a higher fecal than oral relative abundance in half of those subjects, arguing against ectopic colonization in most cases. Interestingly, *D. invisus* was one of the 8 species overlapping between saliva and stool in a shotgun sequencing study (8 healthy adults)^3^ and the only one in a 16S short-amplicon oligotyping-based analysis (>200 healthy adults)^4^ and an analysis using metagenome-assembled genomes (7 healthy adults)^2^. In the only ASV-based, full-length 16S rRNA gene sequencing study of 144 saliva-stool sample pairs collected from Japanese adults^8^, >70% of the subjects had at least one ASV shared between the two niches. The remaining ~30% of subjects had no overlap between their salivary and fecal microbiota. Shared ASVs accounted for a median of ~0.1% of the gut microbiota in each subject, with higher fecal abundances in older subjects and those with dental plaques. The 3 dominant overlapping species were *S. salivarius*, *S. parasanguinis*, and *V. dispar*. Notably, ~40% of the subjects in this study had either hypertension or diabetes, and were thus not considered healthy adults. The present analysis represents the largest one thus far on the subject.

Our methodology was based on data from short sequencing reads. The choice of short vs. long-read methodology depends on the specific question in hand^21^. Metagenomic assemblies of short and long reads tend to produce compositionally similar results. However, while long-read metagenomics recovers a larger fraction of the metagenome with a higher confidence of associated taxonomic annotations, the detection of low-abundance features using long-read approaches at standard sequencing depths is inferior to short-read methods. As oral bacteria were expected *a priori* to have extremely low abundances in the gut, a short-read method was considered to be more suitable for our specific question. The disadvantage of this approach is that we could not map the reads to strains. Rather, we had to resort to clade-specific marker genes presence/absence patterns to indirectly infer same-strain overlaps.

This work is limited by the following: (i) absence of all 4 oral sample types in all 3 studies, (ii) major demographic differences among the studies possibly associated with different diets and lifestyles relevant for microbiota composition, and (iii) the unique population of pregnant women in one study. However, our goal was not a meta-analysis. Rather, we took advantage of the large sample size of a publicly available, curated, metagenomic database to address our question. Finally, sequencing-based methods used in the studies analyzed here do not distinguish between live and dead bacteria, thus an apparent overlap would not necessarily mean the presence of the same live bacteria in different niches. However, this only strengthens our general conclusion and suggests that even the minute overlap found here may not indicate true bacterial overlap.

In conclusion, our findings from secondary analysis of a large, multi-cohort, curated metagenomic database of paired fecal-oral samples including multiple oral sites, species-level taxonomy, and clade-specific marker genes support our previous work and argue against colonization of oral bacteria in the distal gut in healthy adults. This result suggests that finding oral bacteria in the colon may be a marker of pathology, potentially warranting clinical investigation.

## Methods

### Data sources

We used the following criteria to select subjects from curatedMetagenomicData: age ≥18 years, healthy (as defined in each study), at least one stool sample and one paired oral sample (from any site). Because the timing of stool samples could not be pre-planned, they were collected as close as possible to the oral samples, typically within 24 hours. No other inclusion or exclusion criteria were used. We analyzed tables of taxonomic abundances and clade-specific marker genes as provided within curatedMetagenomicData. The authors of curatedMetagenomicData generated these data by downloading the raw sequencing data, without any preprocessing, from all studies and consistently processing them through MetaPhlAn3, yielding species-level taxonomic profiles and coverage across unique, clade-specific marker genes. We used the associated R package, *curatedMetagenomicData*, to query the database.

MetaPhlAn3 was run using default parameters. Specifically, the following parameters were used by the authors of curatedMetagenomicData:

--stat_q (quantile value for the robust average) 0.2: excludes the 20% of markers with the highest abundance as well as the 20% of markers with the lowest abundance

--read_min_len 70 and–min_mapq_val 5: Discards reads shorter than 70 bp and marker hits with a MAPQ value less than 5

--pres_th argument 1.0: minimum number of reads per kilo-base (RPK) to consider a marker present

### Data records

Three studies were eligible for inclusion in our analysis (Table 1). The first study^22^, the Human Microbiome Project, included healthy adults aged 18–40 years in 2 sites in the United States. A lengthy list of criteria was used to ensure lack of evidence of disease in the enrolled subjects. Of all body sites samples (18 in women and 15 in men), saliva, supragingival plaque, buccal mucosa swab, tongue dorsum swab, and fecal samples were relevant for the present work. Each subject provided samples once or twice (within 1 year). Oral samples were collected at least 12 hours after oral hygiene. Stool samples were collected within 24 hours of oral samples. All samples were kept on wet ice until transfer to the freezer, within 4 hours for oral samples. The second study^23^ collected stool, saliva, and skin swab samples (some or all) from Fiji islanders. Paired and stool samples collected from healthy adults were included. Saliva samples were frozen within 30 minutes of collection. Stool samples were collected within 30 minutes of voiding (and within 24 hours of saliva sample collection). The third study^24^ collected stool, tongue dorsum swabs, skin swabs, and vaginal swabs from healthy pregnant women in Italy during or shortly after delivery. Oral and fecal samples were collected from the newborns of those mothers. Samples were frozen immediately. The first two sample types from the mothers were of interest to the present analysis and were included.

**Table 1:** Study characteristics.

	Study #1	Study #2	Study #3
Age, y			n/a
Median (range)	26 (19–39)	50 (20–65)	
Sex, n			n/a
Male	58	50	
Female	54	66	
Sample pairs included, type (n)	ST-SA (5) ST-TD (129) ST-SUPP (118) ST-BM (112)	ST-SA (116)	ST-TD (20)
Collection buffer	ST: none (whole samples) SA: none TD: MoBio buffer SUP: MoBio buffer BM: MoBio buffer	ST: RNA LaterSA: 20% glycerol	ST: n/a TD: SCF-1 buffer
DNA extraction kit	Qiagen	ST: QiagenSA: Maxwell_LEV	n/a
Sequencing platform	IlluminaHiSeq	IlluminaHiSeq	IlluminaHiSeq
Read length	101 bp, paired-end	101 bp, paired-end	100 bp, paired-end
Reads/sample Median (range)	ST: 107,721,245 (20,749,729-238,641,707) SA: 8,279,284 (2,464,625-14,637,415) TD: 90,435,760 (6,872,037-166,408,652) SUP: 56,787,769 (4,192,422-115,781,472) BM: 9,487,939 (483,480-86,589,965)	ST: 106,777,471 (21,379,934-206,522,052) SA: 10,038,935 (3,443,644-96,114,026)	ST: 31,447,612 (3,923,860-173,092,066) TD: 27,756,326 (6,824,785-52,229,053)

BM: buccal mucosa; SA: saliva; ST: stool; SUP: supragingival plaque; TD: tongue dorsum.

### Data analysis

During the assessment of each sample pair type (*e.g*., stool and saliva), we included species that occurred ≥3 times in ≥10% of the samples. No other processing or filtering was performed. We generated heatmaps using the plot_heatmap function of the *phyloseq* package, Bray-Curtis distance, non-metric multidimensional scaling for ordination, and log-transformed species abundances. To plot the shared species, we first selected species that overlapped between the two sample types in ≥10% of sample pairs. We then plotted the frequency of overlap for each of the selected species among all pairs, and separately, the summary statistics for fecal and oral abundances of the overlapping species using the *ggplot2* function. For each species overlapping between each oral niche and stool, we compared oral vs. fecal abundance using a Wilcoxon test and corrected the p values for multiple testing using the Benjamini-Hochberg method^25^. A q value < 0.05 was considered statistically significant.

Clade-specific marker genes corresponding to each overlapping species between the two samples of each pair were then plotted as a presence/absence binary heatmap. To plot these heatmaps, only samples with ≥90% of all marker genes for the species of interest were included. This selection criteria was applied to avoid selecting samples with missing marker genes due to low coverage rather than containing strains with lost marker genes. In this process, we followed a previously used approach, considering that different strains of the same species may contain different subsets of the species-specific marker genes due to gene gain and loss events^3^. Thus, we used specific patterns of marker presence and absence to distinguish between different strains of the same species. We acknowledge that identical marker gene presence/absence patterns do not necessarily indicate the same strain because single nucleotide polymorphisms within the same marker gene could also produce different strains, something that we could not explore in the available database due to the absence of the actual sequence reads. Therefore, our results may overestimate the true overlap, but will not underestimate it. All analyses were performed in R v4.2.0 (R Foundation for statistical Computing, Vienna, Austria).

## Data Availability

R phylogenetic objects and species-specific marker genes derived from curatedMetagenomicData are available in Figshare^26^.

## Code availability

We used curatedMetagenomicData as our sole data source to access data records of interest (https://doi.org/doi:10.18129/B9.bioc.curatedMetagenomicData). R versions 4.3 and later can be used to use this package. All procedures in this package are done by only 4 functions:

(1) curatedMetagenomicData(): used to access the data

(2) mergeData(): used to merge lists of the same data type (e.g. relative abundance data) across studies

(3) returnSamples(): used to return samples across studies sampleMetadata(): used to return sample metadata

The custom R code is available in Figshare^26^.
